# The Role of Virtual Reality in Postural Rehabilitation for Patients with Parkinson’s Disease: A Scoping Review

**DOI:** 10.3390/brainsci15010023

**Published:** 2024-12-29

**Authors:** Francesco Agostini, Marco Conti, Giovanni Morone, Giovanni Iudicelli, Andrea Fisicaro, Alessio Savina, Massimiliano Mangone, Marco Paoloni

**Affiliations:** 1 Department of Anatomical and Histological Sciences, Legal Medicine and Orthopedics, Sapienza University, 00185 Rome, Italy; francesco.agostini@uniroma1.it (F.A.); giovanni.iudicelli@uniroma1.it (G.I.); andrea.fisicaro@uniroma1.it (A.F.); alessio.savina@uniroma1.it (A.S.); massimiliano.mangone@uniroma1.it (M.M.); marco.paoloni@uniroma1.it (M.P.); 2 San Raffaele Institute of Sulmona, 67039 Sulmona, Italy; giovanni.morone@univaq.it; 3 Department of Life, Health and Environmental Sciences, University of L’Aquila, 67100 L’Aquila, Italy

**Keywords:** rehabilitation, virtual reality, Parkinson’s disease, posture

## Abstract

Parkinson’s disease is the second most common neurodegenerative disease worldwide, characterized by bradykinesia, rigidity, tremor, and postural instability. These symptoms often lead to significant postural deformities and an increased risk of falls, severely impacting the quality of life. Conventional rehabilitation methods have shown benefits, but recent advancements suggest that virtual reality (VR) could offer a promising alternative. This scoping review aims to analyze the current literature to evaluate the effectiveness of VR in the postural rehabilitation of patients with PD. A scientific literature search was performed using the following databases: PubMed, PEDro, Cochrane, and Google Scholar, focusing on randomized controlled trials (RCTs) published in English. Our selection criteria included studies that compared VR-based rehabilitation to traditional methods regarding posture-related outcomes. We identified and analyzed nine RCTs that met our inclusion criteria. The results consistently demonstrated that VR-based rehabilitation leads to greater improvements in balance and gait compared to conventional therapy. Key findings include significant enhancements in balance confidence and postural control and a reduction in fall rates. The superior efficacy of VR-based rehabilitation can be attributed to its engaging and immersive nature, which enhances patient motivation and adherence to therapy. VR allows for precise, repeatable training scenarios tailored to individual patient needs, providing a safe environment to practice and improve motor skills. In conclusion, VR-based rehabilitation represents an innovative approach with substantial potential to improve the quality of life for PD patients. However, limitations such as small sample sizes and short intervention durations in existing studies highlight the need for larger multicenter trials with longer follow-up periods to confirm these findings.

## 1. Introduction

### 1.1. Parkinson’s Disease: Pathophysiology, Clinical Features, and Current Treatments

Parkinson’s disease (PD) is the second most common neurodegenerative disease in the world, characterized by bradykinesia, rigidity, tremor, and postural instability [1]. Its incidence increases with age, starting from the age of 50, and affects men more; in 90% of cases, it has sporadic, multifactorial origin, while in 10% of cases, it is genetically determined [2]. The pathogenesis is linked to the selective loss of dopaminergic neurons in the pars compacta of the substantia nigra and in other components of the central nervous system, resulting in motor, vegetative, cognitive, and mood symptoms [3]. The characteristic histological lesions, found on biopsy, are Lewy bodies, cytoplasmic inclusions made up of α-synuclein filaments [4]. In the long term, postural deformities can develop in Parkinson’s patients, such as anterocollis, a forward flexion of the neck, where the chin moves closer to the chest, likely due to dystonia or rigidity in the neck muscles, impairing vision and increasing the risk of falls, as the downward gaze limits awareness of one’s surroundings; camptocormia, a marked forward flexion of the trunk that becomes apparent when standing or walking and improves when lying down, often linked to weakness in the extensor muscles of the spine combined with rigidity; and lateral flexion of the trunk, known as Pisa syndrome, which leads to difficulty in walking, maintaining an upright posture, and performing daily tasks [5]. These lead to progressive postural instability, which is reflected in an increased risk of falls. The risk of falling in Parkinson’s patients is a significant concern due to motor and non-motor symptoms that impair balance, coordination, and reaction times. Falls are a leading cause of injury, disability, and reduced quality of life [6]. The mortality of this pathology is linked to complications resulting from mobility limitations, such as dysphagia and the high number of falls that these patients suffer. The consequences of falls in elderly patients with Parkinson’s disease can be severe and multifaceted, impacting their physical, psychological, and social well-being. Key consequences include fractures, particularly of the hip, wrist, or spine; head injuries, including traumatic brain injuries; and reduced mobility due to injury or fear of further falls; this loss of mobility leads to muscle deconditioning and increases dependence on caregivers. Falls can directly or indirectly contribute to mortality, particularly when associated with head injuries or complications from prolonged immobility, such as pneumonia or pressure ulcers [7,8]. For the clinical staging of PD, the Hoehn and Yahr (HYS) classification is mainly used; it is a simple and widely used system to describe symptom progression. It consists of five stages, starting with mild, unilateral symptoms that do not interfere significantly with daily life. As the disease progresses, symptoms become bilateral or affect the trunk, leading to postural instability. In the later stages, there is increasing dependency as balance, mobility, and daily functioning become more severely impaired [9,10]. Pharmacological therapy makes use of various molecules with proven efficacy, such as levodopa, dopamine agonists, monoamine oxidase (MAO) inhibitors, catechol-O-methyltransferase (COMT) inhibitors, and amantadine [11]. Regarding the rehabilitation aspect, there is a growing body of evidence to support the benefits of therapeutic exercise: regular, targeted exercise can enhance quality of life, slow functional decline, and improve overall well-being [12,13]. Alongside classic physiotherapy methods, more and more innovative therapeutic options are emerging, among which, virtual reality (VR) seems to represent one of the most promising possibilities [14].

### 1.2. Virtual Reality: Types and Functionality

Virtual reality is a technology that creates a simulated environment, allowing users to experience and interact with a digital world that can be similar to the real world. It typically involves the use of screens, visors, and dedicated tools to immerse the user fully in the virtual environment [15]. There are several types of virtual reality: Non-immersive VR: This type includes desktop-based simulations where users interact with a virtual environment through a computer screen using devices like a mouse and keyboard. It is less immersive but still provides a virtual experience. Fully immersive VR: This is the most immersive form, using VR headsets and sometimes additional equipment like gloves or bodysuits to fully engage the user’s senses and provide a lifelike experience. Augmented reality (AR): While not strictly VR, AR overlaps by blending virtual elements with the real world. Users see the real world with digital enhancements overlaid, often through smartphones, tablets, or specialized AR glasses. It is important to clarify that in the literature, exergaming has also been identified as virtual reality [16]. Each type of VR offers different levels of immersion and interaction, catering to various applications from gaming and entertainment to professional training and education.

VR has been shown to influence postural control through several neurophysiological mechanisms, primarily by modulating multisensory integration, visual information processing, and cortical activity. These mechanisms collectively enhance the central nervous system’s ability to adapt and recalibrate balance strategies, making VR a valuable tool in both clinical and research settings.

#### 1.2.1. Multisensory Integration

Postural control relies on the integration of sensory inputs from the visual, vestibular, and somatosensory systems. VR environments can manipulate these inputs, challenging the central nervous system to recalibrate and improve balance. For instance, a study by Kim et al. [17] demonstrated that dynamic virtual environments could induce greater postural disturbances compared to static conditions in stroke patients. This finding suggests that VR’s ability to simulate dynamic perturbations effectively trains the brain’s sensory integration pathways, leading to improved postural responses.

#### 1.2.2. Visual Information Processing

Visual information plays a critical role in maintaining balance, and VR provides a controlled environment to manipulate visual stimuli. A study by Hodgson et al. [18] explored the modulation of visual feedback in both real and virtual environments and its impact on postural control. The findings highlighted that VR-induced neuromodulation could closely mimic real-world balance scenarios, enhancing the integration of sensory inputs and the coordination between sensory systems. This study underscored that visual feedback in VR environments can be precisely adjusted to create challenges or provide support, forcing users to adapt their postural strategies in dynamic ways.

#### 1.2.3. Cortical Activity Modulation

The use of VR in training and rehabilitation has demonstrated the ability to influence cortical activity and modulate postural control. A study by Brock et al. [19] explored the differences in postural control during a visuo-motor task performed in real and virtual environments. The findings suggested that VR influences the complexity and regularity of postural movements, potentially engaging different neural pathways compared to real-world settings. The study highlighted that VR tasks require the integration of visual feedback and motor planning in a way that challenges the central nervous system to adapt and reorganize. These adaptations are linked to changes in cortical activity, as the brain actively processes the mismatched sensory inputs commonly encountered in VR environments. This unique demand on cortical networks may explain the observed improvements in postural control and motor coordination during VR-based rehabilitation.

### 1.3. Virtual Reality and Neurorehabilitation

VR is increasingly recognized as a groundbreaking tool in neurorehabilitation, revolutionizing the way therapies are delivered for various neurological conditions.

For stroke patients, VR has been particularly beneficial. A systematic review by de Rooij and colleagues [20] highlighted that VR training significantly improves balance and gait abilities. By offering a dynamic and stimulating alternative to conventional therapies, VR enables patients to practice essential motor skills in a safe, controlled setting. This is especially important for tasks that mimic real-life challenges, which can be crucial for regaining independence.

VR has shown significant potential in the rehabilitation of patients with multiple sclerosis (MS). A systematic review by Basalic et al. [21] evaluated the effectiveness of VR interventions on balance in individuals with MS. The study concluded that VR-based therapies, especially when compared to standard balance training, consistently improved balance in MS patients. Additionally, the use of robot-assisted technology combined with 2D VR yielded superior results in balance rehabilitation. However, the effects of VR interventions on walking speed varied.

In the realm of pediatric neurorehabilitation, children with cerebral palsy have shown notable improvements in balance and motor control through VR interventions. By incorporating playful and interactive elements, VR transforms therapy into a motivating activity, encouraging children to participate actively and consistently [22].

Additionally, VR has demonstrated remarkable potential in addressing phantom limb pain, a challenging condition experienced by many amputees. Through immersive environments, VR allows patients to visualize and manipulate a virtual representation of their missing limb, helping to reorganize neural circuits and potentially alleviate pain. This innovative approach provides a unique pathway for managing pain and improving quality of life [23].

The purpose of this scoping review is to analyze the literature currently available and look for the most recent evidence that allows us to draw conclusions on the effectiveness of VR in the postural rehabilitation of patients with PD.

## 2. Materials and Methods

### 2.1. Search Strategy

A scientific literature search was performed by two researchers using the following databases: PubMed, PEDro, Cochrane, and Google Scholar. The search was performed using the following MeSH terms: ((rehabilitation AND Parkinson) OR (virtual reality AND Parkinson) OR (exergames AND Parkinson) OR (postural instability AND Parkinson) OR (therapeutic exercise AND Parkinson)). Our search was restricted using the following filters: only randomized controlled trials (RCTs), published in the English language, with full text available, and not published earlier than 2000. The inclusion criteria were the following: RCTs addressing the topic of the use of VR in the postural rehabilitation of patients with PD, evidence obtained from human patients, and articles in the English language. The exclusion criteria were the following: full text not available, RCTs lacking rehabilitative interventions for the postural rehabilitation of patients with PD and/or not providing evidence-based support, and PEDro score inferior to six. The scientific search was conducted in June 2024. It is important to clarify that in the literature, exergaming has also been identified as VR, as evident from Cochrane’s “Virtual reality for stroke rehabilitation”. In recent years, the spread of headsets has allowed for differentiation between immersive and non-immersive VR. It is clear that these different modalities have areas of overlap, but in the near future, it will be important for researchers to better define the technological and therapeutic contents of the adopted treatment. This will allow for better clinical translation of the potential benefits of exergaming, non-immersive VR, and immersive VR, adapting them to the specific functionality of the patient according to the principles of personalized medicine. Additional data are provided in Supplementary Materials (File S1).

### 2.2. Selection Criteria

We evaluated for inclusion RCTs answering the question, “Is virtual reality rehabilitation for Parkinson’s patients as effective as traditional rehabilitation in terms of posture-related outcomes?” Specifically, all RCTs were assessed for eligibility according to the following participants, intervention, comparison, and outcomes (PICO) model [24]: Participants: consisting of patients affected by PD. Intervention: consisting of balance rehabilitation with VR assistance, regardless of the protocol and the type of VR. Comparison: consisting of conventional rehabilitation. Outcome measures: consisting of all balance- and posture-related outcomes. Study design: scoping review. The PICO model’s eligibility criteria are reported in Table 1.

### 2.3. Study Selection

Two reviewers independently screened the articles to evaluate their eligibility (degree of agreement: 93%). Discrepancies and inconsistencies were resolved through discussion and by consulting a third reviewer. When an article was considered eligible, the full text was obtained and independently evaluated by the two reviewers for inclusion. Duplicates were excluded.

### 2.4. Data Extraction

The same reviewers independently performed data extraction, completing a specific preformed form. For each included article, details were extracted on citation, authors, publication year, journal, study design, date of the search, number of total participants, types of intervention, types of virtual reality system, outcomes of the study, and length of follow-up.

### 2.5. Study Quality Assessment

Two authors independently assessed the quality of the individual studies, and discrepancies between the two authors were solved by discussion. The randomized controlled trials included in this review were evaluated using the PEDro scale to assess their methodological quality. The PEDro scale, which includes 11 criteria, is a validated tool commonly used for this purpose, ensuring a rigorous and standardized assessment of the studies [25].

## 3. Results

**Figure 1:**
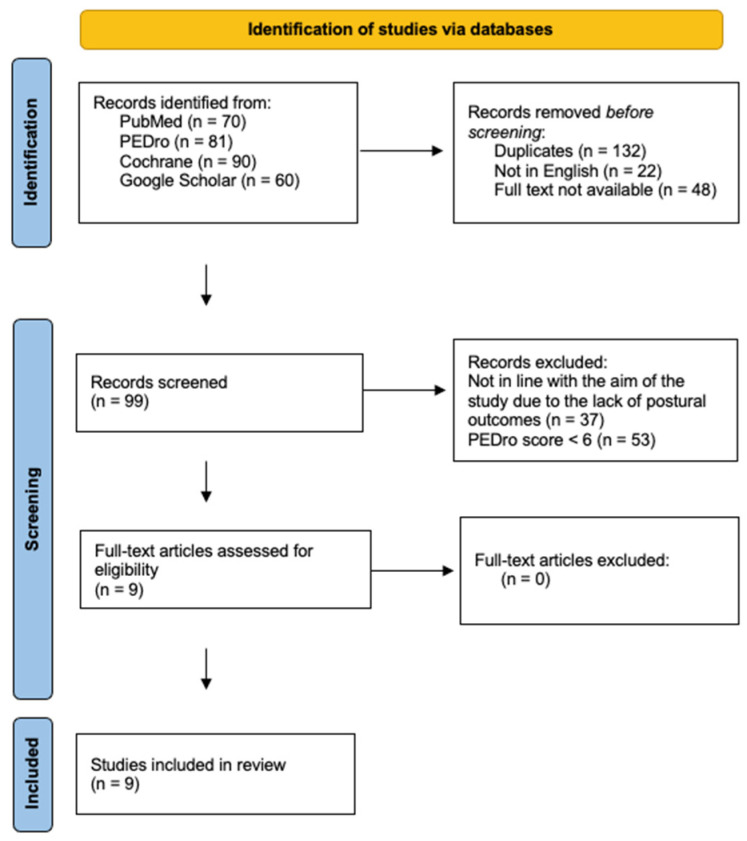
Flowchart.

The search returned a total of 301 studies. After eliminating duplicates and studies for which the full text could not be found, we included a total of nine RCTs that met our inclusion criteria (Figure 1). Each study was evaluated with the PEDro scale, as reported in Table 2. The characteristics of all the studies included in the scoping review are reported in Table 3.

In a study by Yang et al. [26], 23 patients diagnosed with PD were recruited and then divided into an experimental group (11 patients), which underwent rehabilitation with a home-based VR system, and a control group (12 patients), which underwent conventional rehabilitation supervised by a physical therapist. For postural evaluation, the primary outcome used was the Berg Balance Scale, an assessment scale for balance and fall risk based on a score ranging from 0 to 56 points, consisting of 14 items related to the patient’s ability to perform various balance-related tasks; a score below 40 is suggestive of fall risk. As a secondary outcome, gait was assessed using the Dynamic Gait Index, a scale composed of eight items that aim to evaluate the patient’s stability during adaptive gait. Evaluations were conducted before the test and at 6 weeks and 8 weeks from the start of rehabilitation. All sessions were held one hour after taking levodopa during the ON phase; the frequency was twice a week, with each session lasting about 50 min. Patients in the study group performed exercises using a proprietary-software balance board connected to a monitor capable of providing visual and auditory feedback; the center of pressure was used to control virtual objects (e.g., a car) in various games with increasing task difficulty. The control group underwent conventional therapy under the guidance of a physical therapist, with targeted static and dynamic exercises to improve balance and posture. This study demonstrated that both rehabilitation protocols were equally effective in improving patients’ balance and gait, with no significant differences between the two groups. Despite this, the authors believe that the enriched environment of virtual reality and the various stimuli it provides may stimulate the brain more than traditional rehabilitation. Additionally, especially in geographical areas far from rehabilitation centers or services, the ability to perform home-based rehabilitation could be an advantage in terms of service accessibility.

Gandolfi et al. [27] conducted an interesting study by recruiting 76 Parkinson’s patients and randomly assigning them to a control group, which followed conventional rehabilitation in a hospital setting, and an experimental group, which followed an innovative home-based telerehabilitation program with VR, to compare the differences between the two methods in improving postural stability. Both groups followed a program consisting of 50 min exercise sessions three times a week for seven consecutive weeks. The VR group performed exergames at home using the Nintendo Wii Fit platform and a balance board, always under the remote guidance of a physical therapist during the ON phases after taking medication; the control group performed conventional exercises in a clinic, guided by a physical therapist, mainly based on self-destabilization and external destabilization with increasing difficulty. Patients were evaluated before treatment, after treatment, and one month after the end of treatment. For the primary outcome, the Berg Balance Scale was also used in this study, and as a secondary outcome, the Activities-Specific Balance Confidence (ABC) Scale was employed, which evaluates the subjective confidence level perceived by the patient during daily activities, with a score ranging from 0 to 100, where a result below 75.6 suggests an increased fall risk. Significant differences were found for the primary outcome at the 7-week control, with a slight advantage in the VR group, while no differences were found for the secondary ABC outcome. Additionally, the economic costs of supporting these protocols were calculated, highlighting a total savings of about EUR 5000 in the experimental group: Each patient in this group faced an average expense of EUR 383.55, compared to EUR 602 spent by the patients in the control group.

In a study by Maranesi et al. [28], a comparison was made between VR rehabilitation and conventional physiotherapy to evaluate the effects on posture and fall risk. Thirty-two patients were divided into a technology group, which performed the rehabilitation program with a proprietary system called Tymo, consisting of a dynamic platform, and a control group that performed conventional exercises. The primary outcome was evaluated using Tinetti’s Performance-Oriented Mobility Assessment (POMA), a tool used to measure abilities in various balance and gait tasks, indicating a high fall risk for scores below 19 and a minimal fall risk for scores above 25. As a secondary outcome, the Falls Efficacy Scale International (FES-I) was considered, a questionnaire that asks the patient about the perceived fear of falling while performing sixteen activities, where a result of 16 indicates minimal fear, and a maximum result of 64 indicates severe concern. The control group, which lost two patients who did not complete the treatment, performed ten training sessions twice a week, lasting about 50 min each, with breathing exercises, task-oriented exercises, and static and dynamic destabilization and coordination exercises, while the experimental group performed 30 min of traditional therapy combined with 20 min of technological treatment with exergames aimed at postural control and balance, where the patient’s body acted as a joystick to achieve various goals (e.g., picking apples with a basket). Based on the evaluations before and after treatment between the two groups, the technology group achieved a greater improvement in the primary outcome in terms of balance, fall risk, and gait; no significant differences were observed between the two methods for the secondary outcome. It is noteworthy that the technology group also improved in the psychological sphere, measured through the SF-12 Health Survey, a scale that assesses quality of life, suggesting that the virtual experience may be more appreciated than traditional exercise performed in outpatient settings.

Kashif et al. [29] conducted a study on sixty Parkinson’s patients, dividing them into three groups of twenty who received, respectively, VR rehabilitation combined with traditional physical therapy, motor imagery techniques combined with traditional physical therapy, and exclusively traditional therapeutic exercise. The postural outcome was evaluated with the Berg Balance Scale and the Activities-Specific Balance Confidence Scale at the beginning of treatment and at six weeks, twelve weeks, and one month after the end of treatment, always during the ON phase, two hours after medication administration. Each training session was scheduled to last 60 min and be performed three times a week for a total of twelve weeks. Data analysis concluded that the combination of VR and traditional physical therapy proved to be the most effective in improving balance and overall motor functions. The VR platform used was once again the Nintendo Wii gaming platform, which, according to the authors, could stimulate neural plasticity better than traditional therapy while also activating the reward system to motivate the patient throughout the training, maintaining high compliance.

Gulcan et al. [30] conducted a comparative study between two groups of 15 people with Parkinson’s disease, the first treated with VR exercises using the C-Mill VR system combined with conventional therapy and the second group treated exclusively with conventional therapy. The control group performed 60 min sessions three times a week for six weeks, while the experimental group performed 90 min sessions with the same weekly frequency. Patients were evaluated before and after six weeks of intervention. Balance was assessed with the Berg Balance Scale and the Activity-Specific Balance. At the end of the study, the considered outcomes improved in both groups, but without significant differences between the two methods.

Feng et al. [31] compared the effects of VR on a group of 14 patients with the outcomes of a control group with an equal number of participants, who performed conventional physiotherapy. All participants performed 45 min sessions five times a week for 12 weeks, two hours after taking medication. The outcomes considered, evaluated before and after treatment, were measured with the Berg Balance Scale and the Functional Gait Assessment, a 10-item score where the patient is made to walk with progressively increasing difficulty, such as with closed eyes; each item is rated from 0 to 3, where 3 is normal task performance, and the highest possible score is 30. At the end of the treatment, the BBS score improved in both groups, but significantly more in the experimental group (6 points) compared to the control group (2 points); the FGA result also significantly improved compared to the control group, 7 points versus 2.

In a multicenter study by Goffredo et al. [32], 97 patients were recruited and randomly divided into an experimental group that performed home-based telerehabilitation with a VR system and a control group that performed self-administered conventional physiotherapy at home. Postural stability was evaluated before and after the rehabilitation cycle using the Mini-Balance Evaluation System Test, an abbreviated version of the Balance Evaluation System Test composed of 14 items, with a maximum score of 28 points obtainable by correctly performing tasks of postural control, reactivity, and functional gait. Both treatments and evaluations were conducted during the ON periods after medication administration. Patients in both groups performed a total of 30 training sessions lasting 45 min each, 3–5 times a week. The experimental group was trained with the Khymeia tablet system, an Italian Ministry of Health-approved device for patients with neurological disorders that uses inertial sensors to capture patient movements, providing visual and auditory feedback in a simulation environment. A physical therapist collected data weekly to verify patient adherence and modify exercise difficulty parameters if necessary. The control group performed personalized conventional motor activities, monitored through a personal patient diary. At the end of the experiment, all study participants showed improved balance, but the experimental group performing telerehabilitation with VR exhibited a significant score increase, demonstrating that virtual reality was not only effective but also superior to conventional therapy.

Shih et al. [33] explored the impact of VR rehabilitation on postural control and balance in PD patients. The study included 20 participants who were randomized into VR and control groups. The VR group engaged in VR-based postural control exercises, while the control group performed traditional postural exercises. Postural stability was assessed using the limits-of-stability and one-leg stance tests. Balance was assessed using the Berg Balance Scale and the timed up and go test. Both training approaches improved BBS and TUG performance. Additionally, balance exergames training showed significantly greater effectiveness for directional control during the LOS test compared to conventional balance exercises.

A study by Liao et al. [34] demonstrated that 12 sessions of virtual rehabilitation training significantly improved obstacle-crossing performance and dynamic balance in PD patients compared to the results of no structured exercise. The improvements persisted for at least one month. VR training was more effective than traditional exercise in improving movement velocity in the limits-of-stability test, indicating enhanced balance control. The VR group showed greater improvements in dynamic balance and sensory integration, contributing to better obstacle-crossing performance. The significant enhancements in stride length and velocity during obstacle crossing suggest that VR-based training may offer superior benefits for PD patients. The findings support the inclusion of VR training in exercise programs for PD patients, highlighting its potential to improve mobility and reduce fall risk through engaging and effective rehabilitation strategies.

**Table 2:** Eligibility criteria are specified as follows: C1, eligibility criteria were specified; C2, subjects were randomly allocated to groups; C3, allocation was concealed; C4, the groups were similar at baseline regarding the most important prognostic indicators; C5, there was blinding of all subjects; C6, there was blinding of all therapists who administered therapy; C7, there was blinding of all assessors who measured at least one key outcome; C8, measures of at least one key outcome were obtained from more than 85% of the subjects initially allocated to groups; C9, all subjects for whom outcome measures were available received the treatment or control condition as allocated, or where this was not the case, data for at least one key outcome were analyzed by “intention to treat;” C10, the results of between-group statistical comparisons are reported for at least one key outcome; and C11, the study provides both point measures and measures of variability for at least one key outcome.

Article	C1	C2	C3	C4	C5	C6	C7	C8	C9	C10	C11	Total Score
Feng et al. [31]	Yes	Yes	No	Yes	No	No	Yes	Yes	Yes	Yes	Yes	8/10
Gulcan et al. [30]	Yes	Yes	No	Yes	No	No	Yes	Yes	Yes	Yes	Yes	8/10
Gandolfi et al. [27]	Yes	Yes	No	Yes	No	No	Yes	Yes	Yes	Yes	Yes	8/10
Goffredo et al. [32]	Yes	Yes	No	Yes	No	No	Yes	Yes	Yes	Yes	Yes	8/10
Kashif et al. [29]	Yes	Yes	No	Yes	No	No	Yes	Yes	Yes	Yes	Yes	8/10
Liao et al. [34]	Yes	Yes	No	Yes	No	No	Yes	Yes	Yes	Yes	Yes	8/10
Maranesi et al. [28]	Yes	Yes	No	Yes	No	No	No	Yes	Yes	Yes	Yes	7/10
Shih et al. [33]	Yes	Yes	No	Yes	Yes	No	No	Yes	Yes	Yes	Yes	8/10
Yang et al. [26]	Yes	Yes	No	Yes	No	No	Yes	Yes	Yes	Yes	Yes	8/10

**Table 3:** Characteristics of the studies. EG: experimental group. CG: control group. BBS: Berg Balance Scale. DGI: Dynamic Gait Index. MMSE: Mini Mental State Examination. HYS: Hoehn–Yahr stage. ABC: Activities-Specific Balance Confidence. FAC: Functional Ambulation Category. POMA: Performance-Oriented Mobility Assessment. FES-I: Falls Efficacy Scale International. FGA: Functional Gait Assessment. MOCA: Montreal Cognitive Assessment. Mini-BES Test: Mini-Balance Evaluation Systems Test. LOS: limits of stability. OLS: one-leg stance. TUG: timed up and go. OCP: obstacle-crossing performance.

Authors	Population	Inclusion Criteria	Exclusion Criteria	Outcome	Virtual Platform	Results	Follow-Up
Yang et al. [26]	11 EG 12 CG	55–85 years old MMSE > 24 HYS 2–3	Untreated depression Visual or auditory deficits	BBS DGI	Micro-Star International Co.	EG = CG	8 weeks
Gandolfi et al. [27]	38 EG 38 CG	18+ years old HYS 2.5–3	Visual or auditory deficits MMSE < 24 Depression	BBS ABC	Nintendo Wii	BBS: EG > CG ABC: EG = CG	4 weeks
Maranesi et al. [28]	16 EG 16 CG	65+ years old HYS 1–3 FAC ≥ 2	MMSE < 24 Severe depression	Tinetti POMA FES-I	Tymo system	POMA: EG > CG FES-I: EG = CG	5 weeks
Kashif et al. [29]	20 EG 20 CG1 20 CG2	50–80 years old HYS 1–3 Intact cognition	MMSE < 24	BBS ABC	Nintendo Wii	BBS: EG > CG12 ABC: EG > CG12	16 weeks
Gulcan et al. [30]	15 EG 15 CG	40+ years old HYS 1–3	MMSE < 24 Visual or auditory deficits	BBS ABC	Nintendo Wii	BBS: EG = CG ABC: EG = CG	6 weeks
Feng et al. [31]	14 EG 14 CG	50–70 years old HYS 2.5–4	Other causes of tremors Severe comorbidities Visual or auditory deficits	BBS FGA	Not specified	BBS: EG > CG FGA: EG > CG	12 weeks
Goffredo et al. [32]	49 EG 48 CG	<80 years old MOCA > 17	Psychiatric disorders Visual or auditory deficits	Mini-BES Test	Khymeia system	EG > CG	10 weeks
Shih et al. [33]	11 EG 11 CG	HYS 1–3 Stable medication	MMSE < 24 Severe comorbidities	LOS OLS BBS TUG	Kinect	LOS: EG > CG OLS: EG > CG BBS: EG = CG TUG: EG = CG	8 weeks
Liao et al. [34]	12 EG 12 CG1 12 CG2	HYS 1–3 Stable medication Independent gait	MMSE < 24 Past seizure Pacemaker Vision deficits	OCP LOS	Nintendo Wii	OCP: EG > CG12 LOS: EG > CG12	4 weeks

The studies analyzed in this review collectively highlight the potential of VR-based rehabilitation in improving postural control and balance for patients with Parkinson’s disease. Despite variability in protocols, populations, and VR platforms, several consistent patterns emerged:

**Balance and Postural Control**: Most studies reported significant improvements in balance outcomes, with experimental VR groups often outperforming control groups undergoing conventional therapy. For example, immersive systems like those used in the studies by Feng et al. [31] and Goffredo et al. [32] demonstrated greater efficacy in improving scores on balance scales, such as the Berg Balance Scale, compared to that of non-immersive systems. These findings suggest that VR’s engaging and adaptive features may enhance motor learning and balance recovery.

**Dual Benefits of Immersion and Engagement**: Immersive VR systems appear to provide a dual benefit by simultaneously improving physical outcomes (e.g., postural stability) and cognitive functions (e.g., attention and memory). Studies employing digital platforms showed high compliance and patient satisfaction, suggesting that engagement plays a key role in rehabilitation success.

**Home-Based Rehabilitation**: Home-based VR interventions, such as those described by Yang et al. [26] and Goffredo et al. [32], demonstrated comparable or superior results to clinic-based conventional therapy. This indicates that VR can increase accessibility while maintaining therapeutic effectiveness, particularly in remote or underserved areas.

**Variability Across Studies**: While most studies reported positive outcomes, the degree of improvement varied. For example, Gulcan et al. [30] observed no significant differences between VR and conventional groups in balance measures. Such discrepancies may be attributed to differences in intensity, duration, and technologies employed across studies.

The reviewed studies collectively highlight the superior efficacy of VR-based interventions in improving postural control compared to traditional methods. Most studies reported significant improvements in balance scales, with immersive VR showing slightly greater benefits than non-immersive systems.

## 4. Discussion

This scoping review provides compelling evidence that VR-based rehabilitation is not only effective for postural rehabilitation but offers significant benefits over conventional physical therapy for improving balance and postural control in patients with PD.

The superior efficacy of VR-based rehabilitation can be attributed to several factors. First, VR provides a highly engaging and immersive environment that enhances patient motivation and adherence to therapy. This increased engagement likely contributes to the greater improvements observed in balance and gait measures [27,28,29,30,31,32,33]. Second, VR allows for precise and repeatable training scenarios that can be tailored to individual patient needs, facilitating more effective and personalized rehabilitation. The ability to simulate real-world challenges in a controlled environment enables patients to practice and improve their motor skills in a safe setting, thereby reducing the risk of falls and enhancing overall functional mobility [28,29]. In addition to physical improvements, VR-based interventions also offer cognitive benefits. Several studies in this review highlighted enhancements in cognitive functions, such as improved attention and memory, which are crucial for the daily functioning of PD patients. This dual benefit of motor and cognitive improvements underscores the comprehensive potential of VR-based rehabilitation for addressing the multifaceted challenges faced by PD patients. Since PD patients often struggle with dual-task activities (e.g., walking while talking), incorporating these elements into VR training may help improve not only motor function but also cognitive resilience, enhancing patients’ overall functional independence in daily activities [29,30,31]. Furthermore, VR-based rehabilitation provides a valuable alternative for patients in geographically isolated areas, where access to traditional rehabilitation services may be limited. The ability to conduct home-based VR rehabilitation can significantly improve service accessibility and reduce the burden on healthcare facilities [26,27,28,29,30,31,32]. Exergames represent an innovative and customizable tool for rehabilitation, capable of overcoming some of the limitations of traditional physiotherapy. However, the variability in technologies employed and in the application of intervention protocols suggest the need for further research to standardize treatment practices and validate the most effective approaches. Additionally, the use of different software and peripherals could be a confounding factor in interpreting the results. The advantage of the immersive nature of VR has been noted to increase patient motivation and engagement during rehabilitation [28]. However, the long-term adherence to VR-based rehabilitation programs remains underexplored. A common limitation across studies is the small sample size, which reduces the generalizability of findings. Additionally, the variability in VR systems and protocols complicates comparisons and highlights the need for standardized methodologies in future research. Integrating wearable technologies, such as motion sensors or smart textiles, with VR systems could enhance the effectiveness of rehabilitation by providing real-time feedback on a patient’s movements and posture. This combination could allow for more precise monitoring and adjustment of exercises, further improving motor learning and outcomes. Additionally, data collected from wearables could help track progress and customize future rehabilitation sessions. Lastly, VR could reduce the overall costs of rehabilitation, as it does not require expensive peripherals and allows a single physiotherapist to manage multiple patients. One study estimated the cost of rehabilitation with both systems, including the cost of personnel for screening, assessment, and resource utilization; the total cost for rehabilitation per patient was EUR 383.55 for the VR group and EUR 602.1 for the control group [27]. Furthermore, the reduced use of private transportation to reach clinics or outpatient facilities could also have a positive impact on the environment.

We conducted a literature review of existing systematic reviews and meta-analyses on the topic to compare their conclusions with our findings for a comprehensive understanding of VR’s potential. Kashif et al. [35], in 2022, reported significant improvements in balance, gait, and motor skills in PD patients undergoing VR-based rehabilitation compared to traditional methods. Their review highlighted the use of VR for enhancing static balance and showed superior outcomes when combined with physical therapy. However, they called for higher-quality research to address methodological gaps. Our scoping review aligns with these findings by emphasizing the engaging and motivational nature of VR in improving balance. Both reviews underscore the importance of immersive environments in facilitating motor learning and adherence, although Kashif’s findings focus more on methodological variability among studies. Hussain et al. [36] evaluated the impact of Wii Fit-based non-immersive VR exercises on balance and cognition. They found statistically significant improvements in balance but limited effects on cognitive outcomes. Marotta et al. [37] emphasized VR’s role in cognitive rehabilitation for PD, particularly in enhancing executive functions. They found that VR, combined with exergaming, could stimulate neuroplasticity and motor reorganization through interaction with virtual environments. While cognitive benefits were not a primary focus of our scoping review, the findings suggest that immersive VR could indirectly influence cognitive domains. García-López et al. [38] demonstrated the effectiveness of non-immersive VR in reducing fall risks and improving static and dynamic balance. However, they noted no significant differences compared to control groups across all metrics. These results partially contrast with the current scoping review’s conclusion that immersive VR outperforms traditional rehabilitation in most balance-related outcomes. This discrepancy could be due to the varying levels of immersion and technological sophistication in VR systems. Kwon et al. [39] found that VR-based rehabilitation significantly improved balance, as measured by the Berg Balance Scale. However, gait, activities of daily living, and quality of life showed no significant differences compared to outcomes achieved using conventional therapies. Rodríguez-Mansilla et al. [40] underscored VR’s ability to provide sensory feedback, improve adherence, and offer a motivational framework for patients. They concluded that VR is a viable alternative for personalized rehabilitation and home-based treatment. These conclusions strongly align with the current findings. The emphasis on adherence and personalized feedback resonates with this scoping review’s recognition of VR’s engaging and customizable nature. A Cochrane review by Dockx et al. [41] on VR in PD rehabilitation identified low-quality evidence supporting VR’s efficacy for improving gait and balance. It highlighted the need for larger, more rigorous studies to validate these findings. While the Cochrane review called for better evidence, the present scoping review identified more recent studies that consistently report superior balance outcomes with VR, signaling advancements in the field since 2016. All this evidence, consistent with our results, strengthens the position of virtual reality as a tool to be integrated into the rehabilitation process for these patients.

This review highlights several recurring strengths and limitations in the current literature, as outlined below:

**Strengths of VR-Based Interventions:**
  o Efficacy: VR rehabilitation is a valid alternative to conventional physiotherapy
  o Enhanced Engagement and Motivation: VR’s interactive and immersive environments appear to foster higher patient engagement and adherence compared to conventional therapy, as evidenced by high compliance rates reported in several studies.
  o Customizability: VR allows for individualized rehabilitation, offering tailored exercises that adapt to the patient’s abilities and progression. This flexibility may contribute to more effective motor learning and better outcomes.
  o Accessibility: Home-based VR interventions reduce barriers to rehabilitation for patients in remote or underserved areas, offering comparable benefits to clinic-based therapies.
**Limitations of the Current Literature:**
  o Small Sample Sizes: Most studies included small cohorts, limiting the generalizability of findings.
  o Variability in Protocols and Technologies: Differences in the type of VR systems (immersive vs. non-immersive), intervention protocols, and outcome measures make it challenging to draw definitive conclusions about best practices.
  o Short Intervention Durations: Many studies employed short rehabilitation periods, often less than 12 weeks, without long-term follow-up to assess the durability of improvements.

## 5. Conclusions

The studies analyzed demonstrate that VR can offer a rich and stimulating environment capable of improving motor learning and confidence in patients’ daily activities and consequently postural attitude while reducing the risk of falls. A secondary but no less important aspect is the greater engagement that VR can induce in patients, a factor that, especially in the long term, can increase patient compliance. The richness of stimuli provided by this enhanced digital environment and the multiple feedback mechanisms it offers, whether visual, proprioceptive, vibratory, or auditory, may together stimulate neuroplasticity more effectively than conventional exercise. However, a portion of the current older population may be averse to using digital systems, a barrier that will resolve itself as the current younger populations, who have grown up with digital gaming platforms and are therefore more accustomed to their use, eventually face this challenge. Another advantage of VR, particularly related to its use in telerehabilitation, is the ability to reach patients at home, in areas or family situations where going to a clinic or an outpatient facility might be difficult. In our opinion, rehabilitators should consider integrating VR into the rehabilitation programs of patients with PD to offer innovative and stimulating treatments without losing effectiveness, whether independently or alongside conventional therapies. A significant limitation of this review is the heterogeneity of the methodologies and virtual platforms used, making it difficult to establish clear standards for rehabilitation. Additionally, most studies involved small sample sizes, which limits the robustness of the conclusions. Another limitation is the lack of long-term follow-up in the studies, which makes it challenging to assess the durability of the observed improvements. Future research should aim to standardize the use of VR platforms and explore their long-term effects on postural control and other motor outcomes in diverse patient populations. Studies with larger sample sizes and rigorous methodological designs are needed to confirm the observed benefits and establish guidelines for integrating VR into standard clinical practice. Additionally, research should investigate how different patient characteristics, such as age and disease severity, influence the effectiveness of VR-based rehabilitation.

## Figures and Tables

**Table 1:** PICO model eligibility criteria.

Criterion	Details
Population (P)	Patients diagnosed with Parkinson’s disease (PD).
Intervention (I)	Rehabilitation involving virtual reality (VR), regardless of protocol or VR type.
Comparison (C)	Conventional rehabilitation methods or no intervention.
Outcome (O)	Balance, postural control.
Study design (S)	Randomized controlled trials (RCTs) published in English with full text available.
Inclusion criteria	Studies providing evidence of VR interventions in postural rehabilitation for PD patients.
Exclusion criteria	Studies without rehabilitative interventions, studies not providing evidence-based support, full text unavailable, PEDro score < 6.

## Data Availability

All data are reported in the manuscript.

## Supplementary Materials

The following supporting information can be downloaded at: https://www.mdpi.com/article/10.3390/brainsci15010023/s1, File S1: manuscript supplementary.
