# Supplementary material for: The Role of Virtual Reality in Postural Rehabilitation for Patients with Parkinson’s Disease: A Scoping Review
Source: Brain Sci. 2024 Dec 29;15(1):23. doi: 10.3390/brainsci15010023 (PMC11764033; doi:10.3390/brainsci15010023)
Supplement: Supplementary file 1 [file brainsci-15-00023-s001.zip › brainsci-3350131-supplementary.pdf]

https://doi.org/10.17605/OSF.IO/W2VSN
